# Is binding decline the main source of the ageing effect on prospective memory? A ride in a virtual town

**DOI:** 10.1080/20009011.2017.1304610

**Published:** 2017-04-10

**Authors:** Grégory Lecouvey, Julie Gonneaud, Pascale Piolino, Sophie Madeleine, Eric Orriols, Philippe Fleury, Francis Eustache, Béatrice Desgranges

**Affiliations:** ^a^ Normandie Univ, UNICAEN, PSL Research University, EPHE, INSERM, U1077, CHU de Caen, Neuropsychologie et Imagerie de la Mémoire Humaine, 14000 Caen, France; ^b^ Laboratoire Mémoire et Cognition, Institut de Psychologie & Centre de Psychiatrie et Neurosciences INSERM S894, Université Paris Descartes, Paris, France; ^c^ Normandie Univ, UNICAEN, CIREVE, Caen, France

**Keywords:** Intentions, healthy ageing, virtual reality, episodic memory, executive functions

## Abstract

**Objective**: This study was designed to improve our understanding of *prospective memory* (PM) changes in ageing, and to identify the cognitive correlates of PM decline, using a virtual environment, to provide a more realistic assessment than traditional laboratory tasks.

**Design**: Thirty-five young and 29 older individuals exposed to a virtual town were asked to recall three event-based intentions with a strong link between prospective and retrospective components, three event-based intentions with a weak link, and three time-based intentions. They also underwent retrospective episodic memory, executive functions, binding in working memory, processing speed, and time estimation assessments.

**Results**: Older individuals recalled fewer intentions than young adults. While age-related PM decline affected the recall of both prospective and retrospective components, the recall of the latter seemed more challenging for older individuals when the link was weak. This PM decline was linked to an age-related decline in the binding process in working memory, as well as in processing speed, executive functioning, and episodic memory, depending on the nature of intentions.

**Conclusion**: PM appears to be sensitive to ageing, even when the device is thought to be ecological. This decline is particularly pronounced when controlled processes are needed.

## Introduction

*Prospective memory* (PM) refers to our ability to remember to fulfil intentions at some predefined point in the future. Intentions are generally divided into two components: a *prospective* component that involves remembering at the right moment that something has to be done, and a *retrospective* component that refers to the action that has to be done [1]. Intentions are also defined according to the nature of the trigger of the retrieval. For *event-based* (EB) intentions, the retrieval is triggered by the appearance of an external cue in the environment (i.e. take the cookies out of the oven when the timer rings), whereas for *time-based* (TB) intentions, the retrieval is triggered by the occurrence of a target time (i.e. take the cookies out of the oven in 30 minutes). The recall of TB intentions is generally regarded as more complex than the recall of EB intentions as it requires greater involvement of self-initiated processes [2–5].

Over the past 30 years, the effect of ageing on PM has received increasing attention from researchers. Healthy older individuals generally report more prospective than retrospective failures in their daily lives [6]. Nevertheless, experimental studies have yielded inconsistent results, and the effect of ageing on PM remains a complex and unresolved puzzle.

First, most studies suggest that the recall of TB intentions is more affected by ageing than the recall of EB ones [4,7–9]. These results are in line with Craik’s claim [10] that the more self-initiated processes are required to complete a memory task and the less it benefits from an environmental support, the more sensitive the task is to ageing. This stronger age-related decline for the recall of TB intentions has notably been attributed to the difficulty that older individuals have monitoring the clock, owing to an age-related decline in executive functions, attentional resources, and time-estimation abilities [2,4,11]. However, a few studies have reported the reverse pattern, with older adults having greater difficulty recalling EB than TB intentions. These contradictory results have been attributed to the characteristics of the PM tasks such as generous time limits for responding to the ongoing task, giving older individuals enough time to efficiently monitor the passage of time [12], or very challenging EB intentions making recall particularly difficult for older individuals [13].

Beyond the classic distinction between EB and TB intentions, inconsistencies regarding PM’s sensitivity to ageing may be explained by the diversity of processes involved in the retrieval of intentions. *Multiprocess theory* proposes that the retrieval of EB intentions relies either on automatic or on controlled processes, depending on the characteristics of the PM task [8,14–16]. Automatic processes are more likely to be involved when few attentional resources are devoted to the ongoing activity [17–20], and when the prospective cue is *focal* (i.e. when its detection relies on the same processes as the ongoing task [21–23]) or *distinctive* (i.e. when it is easily detectable within the environment [24–26]). Under such circumstances, older individuals generally perform just as well as young ones. When more attentional resources are needed to the ongoing task, and when the prospective cue is nonfocal and less distinctive, studies generally show an age-related decline in the recall of EB intentions (see [27,28] for meta-analyses). The stronger sensitivity of PM to ageing under such circumstances has been related to the age-related decline in controlled processes [15].

Experimental settings have also been found to lead to inconsistencies in the reported effects of ageing on the recall of intentions, with an age-related decline observed in the laboratory, but older adults outperforming their young counterparts in natural conditions [29,30]. Several hypotheses have been proposed to explain this apparent *paradox* [31]. First, it has been argued that older individuals are more likely to use mnemonic strategies to succeed in naturalistic PM tasks because they are more experienced with real-life PM tasks [32]. The same authors suggested that older individuals are more aware of their potential memory lapses. This metacognitive awareness leads them to develop compensatory strategies, such as the use of memory aids (e.g. reminders), which is not possible in laboratory settings. The age–PM paradox has also been attributed to older individuals’ lower ongoing absorption in daily-life activities, and their greater sensitivity to the cost of the ongoing task in laboratory paradigms [33]. Some studies have also suggested that older individuals outperform young participants in naturalistic settings because they are more motivated to complete the task. The disappearance of this surprising age effect when young participants are motivated by incentives argues in favour of this hypothesis [34]. Finally, Schnitzspahn and collaborators [35] suggested that the age-related benefits for PM performance observed in real life are specific to the recall of health-related and social intentions. Over and above all these hypotheses, the age–PM paradox seems to be the consequence of a lack of experimental control in naturalistic settings and a lack of ecological validity in laboratory paradigms [33]. In other words, these authors suggested that the most critical factor in determining the direction of age effects on PM is the task setting rather than the use of more familiar contexts in naturalistic settings. In order to test this hypothesis, Hering and collaborators [36] used a paradigm with high ecological validity and experimental control. They asked young and older individuals to complete the Dresden Breakfast task that consisted in preparing a meal and comprised subtasks including the recall of EB and TB intentions. This study showed that in a real-life situation proposed in the laboratory, young adults completed more subtasks and recalled more EB and TB intentions than older individuals.

The emergence of virtual reality (VR) in the field of neuropsychology also represents an attempt to circumvent the biases of classic assessments. The use of an immersive virtual environment may be a good compromise, as it allows complex naturalistic situations to be reproduced, all the while maintaining a high level of experimental control [37]. This technique has already been conclusively used to assess memory, executive functions, and attention, as well as instrumental activities of daily life such as cooking and driving [38,39]. VR has also been used to assess PM in patients with brain injury [40–42], chronic fatigue syndrome [43], or under the influence of alcohol or drugs [44–46]. From a more fundamental perspective, VR has also helped to highlight the dynamic allocation of cognitive and cerebral processes in PM functioning [47,48]. Kalpouzos and collaborators [48] asked young individuals to recall 22 EB intentions in a familiar virtual environment in which they could move with a joystick. The originality of this study was to combine VR and functional magnetic resonance imaging (fMRI). Results highlighted the recruitment of occipital regions during the search and detection of the prospective cue, suggesting the involvement of the visuoperceptual system. Activation of the frontal eye field and superior parietal cortex was found to subtend top-down visual-attentional cognitive processes before and after the detection of the cue, in order to keep the intention in mind. While the action was being performed, the authors showed that activation switched to frontal and temporoparietal regions including the hippocampus, signalling the involvement of episodic memory in the search for the intention in the mind. Trawley et al. [47] also adopted an innovative methodology based on a virtual environment representing a shopping mall in which participants moved with a mouse and a keyboard to perform a series of purchases (Edinburgh Virtual Errands Task). Prior to the start of the recall, participants were asked to indicate the order they considered optimal to complete all the purchases. Results showed a correlation between PM scores and the ability of subjects to follow their initial plan underlying the importance of planning in PM functioning.

VR has yet to be used to assess the effect of ageing on PM, but three studies have employed a similar methodology. In one of these studies, participants were asked to watch a video showing a person walking through shopping streets [49]. They had to recall 30 EB intentions whenever the different events appeared in the video. In two other studies, PM was assessed in an environment made up of photos of a shopping street [50,51] in which participants were asked to perform a list of actions while moving along the street, by scrolling through the photos using a computer keyboard. All these studies highlighted an age-related decline in PM that was more pronounced for the recall of the retrospective component than of the prospective one. Although these studies used realistic settings, we can assume that participants’ interactions with the environment did not completely satisfy the criterion of immersion that defines a VR task [52].

Studies investigating the cognitive substrates of PM highlighted the involvement of executive functions in the recall of the prospective component of EB and TB intentions. The *preparatory attentional and memory processes* (PAM) [53,54] theory argues notably that successful recall of the prospective component requires the involvement of controlled processes, such as (i) shifting from processes related to the ongoing task to processes related to the monitoring of the environment to detect the PM cues or the target time, and to those involved in PM retrieval, and (ii) inhibition of the ongoing task in order to check the PM cue or target time and to complete the PM one. Updating, planning, and processing speed have also been reported to be involved in the recall of this component [55,56]. Interestingly, one study demonstrated that inhibition and shifting were better predictors of PM performance in ageing than other executive functions and processing speed [57]. Nonetheless, this study did not separately assess the recall of the prospective and retrospective components, which is indispensable because the retrievals of both components do not rely on the same processes.

The retrieval of the retrospective component has been described as primarily requiring retrospective episodic memory [58]. Moreover, *reflexive-associative theory* [59] states that the strength of the link between the prospective cue and the retrospective component influences the degree of involvement of automatic and controlled processes in the retrieval of intentions. If the link is sufficiently strong, the mere perception of the cue reflexively triggers the retrieval of the retrospective component. By contrast, if the link is weak, retrieval of the retrospective component requires the involvement of controlled processes. In line with this theory, studies have shown that individuals can benefit from a strong semantic association between the prospective cue and the retrospective component [59–64]. Moreover, this benefit increases when the association is voluntarily reinforced at encoding [60]. More generally, the encoding and retrieval of both components relies on the encoding and retrieval of associations between them. Baddeley suggested that the encoding and retrieval of associations in memory involves the binding process, which occurs within the episodic buffer of working memory [65]. This process allows for the creation and reinforcement of links between different elements to create a unitary representation in working memory that can then be integrated in long-term memory. Studies suggested that, disturbing the creation of associations, binding decline may be one of the major contributing factors to older adults’ episodic memory decline [66,67]. Interestingly, Gonneaud and colleagues [13] highlighted that binding decline mediates older individuals’ difficulties in retrieving EB intentions. Even if this study did not show such mediation for the recall of TB intentions, we hypothesized that this binding decline also disturbs older adults’ ability to recall TB intentions, by preventing the enhancement of the relationship between the prospective cue (i.e. target time) and the action to perform, that is generally weak.

Despite the interesting results reported by these studies, the exact nature of the cognitive processes involved in the age-related PM decline remains unclear. In a process-oriented view, understanding which processes are involved in the age-related decline when recalling each component of intentions remains one of the keys to disentangling the effects of ageing on PM.

In the present study, we set ourselves four main objectives to clarify the effect of ageing on PM:

1. Assess the effect of ageing on PM using a VR environment to bypass the conditions that lead to the age–PM paradox and improve current understanding of the effect of ageing on the recall of the prospective and the retrospective components of EB and TB intentions. We hypothesized that young individuals would outperform their older counterparts in the recall of the prospective and retrospective components of EB intentions with a strong link between the prospective component and the retrospective component (Link-EB intentions), EB intentions with a weak link between these elements (NoLink-EB intentions), and TB intentions.
2. Investigate the differential effect of ageing on the recall of the prospective component of EB intentions, for which a prospective cue is provided by the environment, and TB intentions, for which individuals have to self-initiate the monitoring of the prospective cue (i.e. a clock). We expected the recall of the prospective component for TB intentions to be more sensitive to ageing than that of the prospective component for Link-EB and NoLink-EB intentions, as more controlled processes are needed to engage in self-initiated monitoring of the clock.
3. Test the influence of the strength of the link between the prospective cue and the retrospective component on the effect of ageing on the recall of the retrospective component of intentions. We expected the recall of the retrospective component to be more sensitive to ageing for both NoLink-EB and TB intentions than for Link-EB intentions as controlled processes have to be engaged in order to voluntary search the information in memory when the association is weak.
4. Ascertain whether the deleterious effect of ageing on binding, executive functions, and retrospective memory accounts for most of the age-related decline in PM. We assumed that the decline of binding in ageing would affect the recall of NoLink-EB and TB intentions in older individuals because it provides the essential glue that sticks the prospective component/retrospective component dyad together. We also expected the age-related decline in executive functions to affect recall of the prospective component of TB intentions and recall of the retrospective component of NoLink-EB and TB intentions, because controlled processes are required for these types of recall. Furthermore, we assumed that the age-related decline in the recall of the retrospective component for No-LinkEB and TB intentions could be explained by an age-related decline in retrospective memory. Finally, a decline in time-estimation abilities could be specifically involved in the age-related decline in the recall of TB intentions.

## Material and methods

### Participants

Thirty-five young and 34 older participants were included in this study. All participants had normal or corrected-to-normal vision, were native French speakers, and had at least 7 years’ schooling. They all held a driver’s license and were active drivers at the time of the experiment. We ensured that they did not have any history of neurological or psychiatric disorders. The older participants were screened for cognitive deficit with the Mattis Dementia Rating Scale [68]. Only individuals who had a normal score (i.e. >137) were included in the study. All participants also underwent the Beck Depression Inventory to exclude those with depressive symptoms (i.e. >6). Of the 34 older participants, five failed to complete the VR task because of discomfort (i.e. dizziness, nausea) with the equipment. As a result, analyses were conducted on 35 young and 29 older individuals (see Table 1), who did not differ in terms of years of schooling and sex ratio. The study was approved by the regional ethics committee (CCP Nord Ouest III).

**Table 1.** Sample characteristics.

	Young Group	Older Group
Age (± SD)	24.80 ± 5.70	65.28 ± 7.49
Years of schooling (± SD)	14.17 ± 2.39	12.86 ± 3.40
Mattis (± SD)		141.24 ± 2.01
Sex ratio (women/men)	12/23	14/15

SD = standard deviation.

### Materials

Participants were tested individually in two separate sessions: one for the VR assessment, the other for the complementary cognitive assessment.

#### Virtual environment

**Figure 1.**
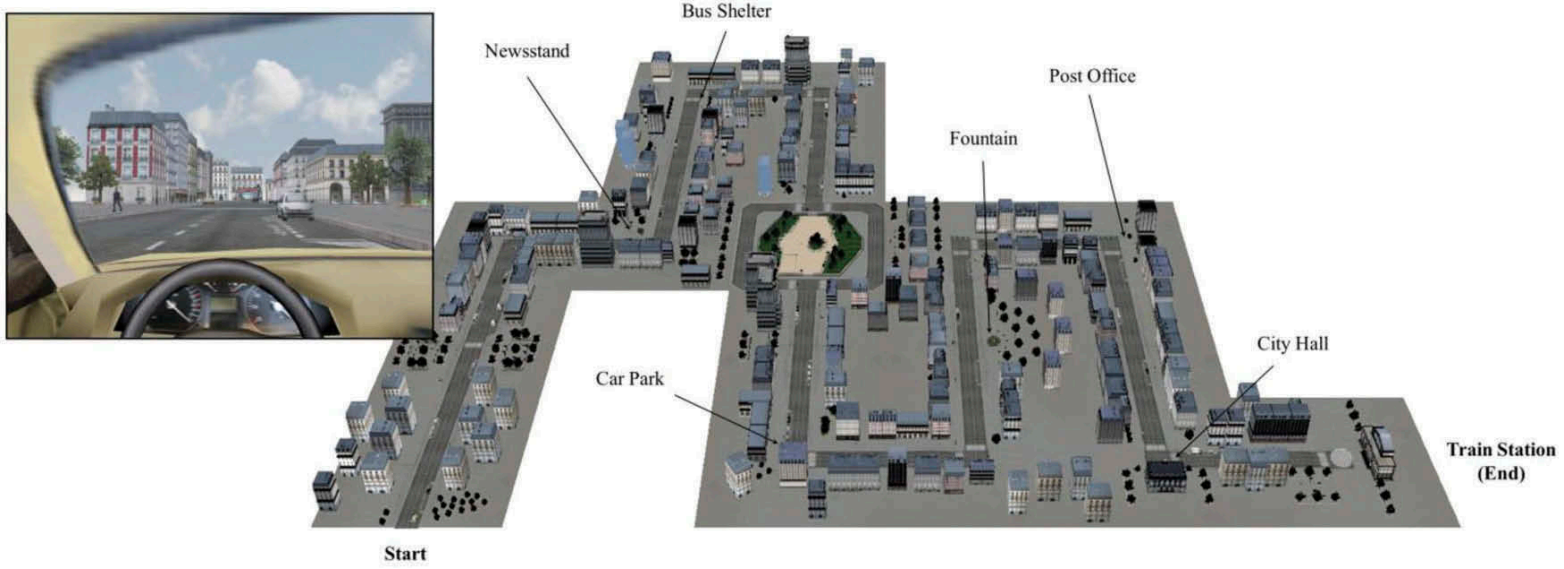
Illustration of the virtual environment including a view from the virtual driving seat during the experiment (left) and a map of the city (right) showing the location of the prospective cues.

The three-dimensional immersive environment was an urban one inspired by buildings in Paris, France. It was created using original software (EditoMem and SimulaMem) developed by the Memory and Cognition laboratory of Paris Descartes University with Virtools Dev 4.0 (see also [69–72]). Participants had to navigate in the environment while driving a virtual car (see Figure 1) using a real steering wheel and gas/brake pedals. The VR sessions took place in a VR-dedicated room at the Interdisciplinary Centre for Virtual Reality (CIREVE) in Caen, France. The virtual environment was projected onto a 180 cm × 240 cm widescreen. Each participant was tested individually and seated approximately 300 cm from the widescreen.

Two cities were created for the purpose of this study. The first one was used to familiarize participants with the device. It was a simple and neutral city featuring ordinary buildings, a few trees, and interactive traffic lights to train participants to use the pedals. There was only one road which formed a loop to enable participants to navigate in the environment.

The second environment was used for the PM stages of the experiment (see Figure 1 for a map of this virtual city). This city had only one possible route, leading to the train station (which signalled the end of the experiment), to minimize the involvement of spatial memory and orientation abilities. The city included a variety of buildings, traffic lights, stores, trees, hoardings, parked cars, and pedestrians. In particular, there were a bus stop, a newspaper kiosk, a parking lot, a fountain, a post office, and a city hall, which were all used as prospective cues for EB intentions. They were located so that participants could easily detect them (i.e. facing the participants in the turns or standing out along the road). In addition to the visual environment, an auditory urban background was added to improve participants’ immersion and sense of presence during the experiment. Finally, an external clock was positioned in such a way that they had to turn their head to monitor the passage of time.

#### Procedure of the PM task

At the beginning of the session, participants underwent a familiarization phase in the first city to avoid the subsequent emergence of difficulties using the device during the PM task. They were informed that the only objective was to learn how to use the wheel and the pedals. They could drive around the city as long as they needed to feel fully confident with the device.

An initial immersion in the city of interest was intended to ensure that participants would easily detect the prospective cues during the PM task. They were asked to drive along the street and pay attention to their surroundings. They were also asked to comply with all French road traffic regulations. They had all the time they needed to drive through this city.

At the end of this initial immersion, we administered a recognition test. Participants had to pick out 14 features of the city from 22 screenshots. Eight of these features were distractor items (i.e. new items) and six were the prospective cues for the forthcoming EB intentions. Feedback was given to participants, and the location of any item that was not recognized was shown on a paper map of the city.

#### PM task

After the familiarization phase, participants were informed that they would again be immersed in the city of interest. They would have to pick a friend up at the train station (i.e. at the end of the route through this city) and fulfil several intentions along the way.

They were provided with a list of nine intentions (see Appendix for a full presentation) on a laptop screen, each displayed one at a time for 10 seconds. There were six EB intentions, half of which (Link-EB) had a strong link between the prospective cue and retrospective component (e.g. buy a book of stamps at the post office), and half (NoLink-EB) had a weak link (e.g. buy glasses at the fountain). The three remaining intentions were TB (e.g. take medication after 4 min). To ensure that all the intentions were correctly encoded, a cued recall test was administered immediately after the presentation of the intentions (e.g. ‘What will you have to do at the post office?’), and any unrecalled intention was repeated until it was correctly encoded. Had any participant needed more than three repetitions to learn an intention, we would have stopped the experiment and discarded the data, but this did not happen.

There was a 10 minute interval between the encoding and retrieval of the intentions, filled by the completion of questionnaires.

After this interval, participants were immersed in the city. The ongoing task was to navigate through the city as far as the train station while paying attention to the virtual environment. Participants were reminded that they would have to pick a friend up at the train station and fulfil several intentions along the way. To fulfil an intention, they were asked to stop the car at the appropriate time or place and orally report the action to perform. For TB intentions, they had a clock to monitor the passage of time. Correct recall was scored two points. One point was awarded if participants stopped the car at the appropriate time or place (prospective component score), and one point if they recalled the correct action (retrospective component score). Each condition had a maximum score of six points. The maximum overall PM score was 18 points.

At the end of the experiment, participants were submitted to a free recall of all intentions. A cued-recall of the missing intentions was also proposed when necessary.

#### Complementary neuropsychological assessment

In order to identify the cognitive correlates of PM decline in ageing, we administered a supplementary cognitive assessment featuring tasks of retrospective episodic memory, processing speed, executive functions, binding in working memory, and time estimation.

Retrospective episodic memory was assessed with the RL-RI16 [73,74]. Participants had to learn a list of 16 words. After semantically encoding the words, they were given 2 minutes to freely recall as many words as they could. Cued recall of the forgotten words followed. A total of three free and cued recalls were administered, followed by a recognition task. Finally, a delayed free and cued recalls were proposed after a 20 minute interval. Participants’ performance was scored as the sum of the words correctly recalled in the free recalls (three free recalls plus delayed free recall/64).

The executive functions assessment focused on inhibition, updating, shifting, and planning. *Inhibition* was assessed using the Stroop test [75]. Participants had to name the colour of 100 coloured rectangles (colour condition). Second, they had to read 100 written names of colours printed in black (reading condition) as quickly as possible. Third, in the interference condition, they were asked to name the colour of the ink in which 100 incongruent colour names were printed. They were instructed to complete each part of the task as quickly as possible. The inhibition score corresponded to the slowdown in the completion time for the third part of the task in comparison with the first one ([Colour time − Interference time]/Interference time) [76].

*Updating* was assessed using the Running Span test [77]. Sixteen consonant strings of varying lengths (4, 6, 8, or 10 letters) were read out. Without prior knowledge of the length of the strings of consonants, participants were required to recall the *n* most recent items in the correct order (*n* ranged from 3 to 6 letters and was adapted to the digit span of each participant). The updating score corresponded to the number of correct trials out of 16.

*Shifting* was assessed using the Trail Making Test (TMT; Army Individual Test Battery, 1944). In Part A, participants had to sequentially link digits as quickly as possible. In Part B, they were required to shift between two sets of stimuli (letters and digits). The shifting score corresponded to the slowdown in the completion time for B in comparison with Part A ([TMTB time – TMTA time]/TMTA time).

*Planning* was assessed using the first part of the Zoo Map test [78]. Participants were given a map of a zoo and instructed to plan the order in which they would visit designated locations, respecting a list of rules. The score (out of 8) reflected planning accuracy, taking into account both the correct order of the tour and violations of the rules.

Processing speed was assessed with the first part of the Stroop test (see above). The score was the time it took participants to name the 100 coloured rectangles.

*Binding* in working memory was assessed using a multimodal integration task (see [77,79,80] for a full description). Four coloured uppercase consonants were displayed in the centre of a 5 × 4 grid. Four coloured crosses were placed randomly in the remaining squares. Participants had to mentally associate the four consonants with the four locations represented by the crosses according to their colour, and maintain this association for one second. They were then provided with a grid featuring a single lowercase letter printed in black inside a square. Participants had to decide whether this letter-location association matched the one they had previously learned. The score was the number of correct trials out of 20.

The *time estimation* assessment was inspired by the one devised by Rueda and Schmitter-Edgecombe [81]. Strings of letters were displayed one by one on a laptop screen for various durations. Participants were asked to read the letters out aloud and estimate the length of the string in seconds. To avoid a counting strategy, the letters appeared at irregular time intervals. Blocks lasted 15, 30, or 40 seconds. The score corresponded to the mean percentage of deviation from the correct time. For each trial, we calculated the difference in seconds between the actual time of the series and the answer of the participant. The difference (i.e. deviation) was then divided by the actual time of the series and multiplied by 100 to obtain a percentage of deviation. Then this percentage of deviation was averaged across all the trials. Time estimation scores were only available for 25 young and 22 older adults.

### Statistical analyses

We assessed the effect of ageing on PM through a 2 (group: young *versus* older) × 2 (intention component: prospective component *versus* retrospective component) × 3 (nature of the intention: Link-EB *versus* NoLink-EB *versus* TB) analysis of variance (ANOVA). Group was entered as a between-participants factor, and intention component and nature of the intention were entered as within-participants factors. We did not investigate second order interactions because they were unnecessary to achieve the objectives of this study. The third order interaction was not significant. However, given our first three objectives, we conducted Tukey’s HSD post-hoc tests to pinpoint specific effects.

In order to test whether the decline in other cognitive functions could account for the age-related PM decline, we first assessed the sensitivity of complementary cognitive tests to age using two-sample *t*-tests including group as a between-participants factor. Forward stepwise regressions were then conducted to identify the best predictors of PM scores among the cognitive measures, including only those for which an age-related decline was found. In order to benefit from sufficient variability in each PM score, which is a prerequisite for conducting such regression analyses, we computed global sub scores. More specifically, forward stepwise regressions were calculated for the scores of the Link-EB, NoLink-EB and TB conditions (i.e. prospective component and retrospective component recall scores were summed), and for the scores of the prospective and retrospective components (i.e. Link-EB, NoLink-EB, and TB intentions recall scores were summed).

Finally, mediation analyses were performed to determine whether the effect of ageing on the recall of intentions was mediated by the effect of ageing on other cognitive functions. We followed the procedure developed by Baron and Kenny [82]. In order to conclude that there was mediation, three regression equations had to be assessed, and the following conditions met: (a) a significant relationship exists between age and the mediator/cognitive function (simple regression); (b) a significant relationship exists between age and PM (simple regression); (c) a significant relationship exists between the mediator/cognitive function and PM (simple regression); and finally (d) the relationship between age and PM must decrease when the mediator/cognitive function is included in the regression model (multiple regression). If the relationship in (d) becomes nonsignificant, the mediation is complete. The bootstrapping method with bias-corrected confidence estimates was used to validate the significance of the mediation [83,84]. A 95% confidence interval (CI) of the indirect effects was obtained with 5000 bootstrap resamples using PROCESS, implemented in SPSS (http://www.processmacro.org/), which is an ordinary least squares regression-based path analytic framework. The 95% CI obtained with this method must not contain zero for there to be a significant effect of mediation.

The significance threshold was set at *p* < .05.

## Results

### Effect of ageing on PM performance

The ANOVA revealed a significant main effect of group on PM performance (*F*(1,62) = 46.41, *p *< .001, η^2^ = .43), with young individuals recalling more intentions than older ones. Young individuals outperformed their older counterparts for the recall of both the prospective and retrospective components of Link-EB (*p* < .001 and *p* < .001), NoLink-EB (*p* = .002 and *p* < .001), and TB intentions (*p* < .001 and *p* < .001).

The third order interaction was not significant (*F*(1,124) = 1.819, *p *= .17). However, as we expected a greater effect of age on recall of the prospective component for TB intentions (i.e. more involvement of self-initiated processes) and on recall of the retrospective component for NoLink-EB and TB intentions (i.e. no reflexive retrieval of the intention due to a weak strength of association), we conducted post hoc analyses to pinpoint specific effects.

### Comparison of the recall of the prospective components of Link-EB, NoLink-EB, and TB intentions in young and older participants

In young participants, there was no difference between the recall of prospective component of Link-EB and NoLink-EB intentions (*p *= .13) and NoLink-EB and TB intentions (*p *= .65). However, they performed better when recalling the prospective component of Link-EB than TB intentions (*p *= .05). In older participants, there was no difference between the recall of the prospective component of Link-EB and NoLink-EB intentions (*p *= .74), Link-EB and TB intentions (*p *= .31), and NoLink-EB and TB intentions (*p *= .18).

### Comparison of the recall of the prospective versus retrospective components of Link-EB, NoLink-EB, and TB intentions in young and older participants

While young individuals recalled both components of Link-EB intentions, NoLink-EB intentions, and TB intentions equally well (*p *= .75, *p *= .09, *p *= .13, respectively), older individuals only recalled both components equally well for Link-EB intentions (*p *= .31). They recalled more efficiently the prospective than the retrospective component for NoLink-EB intentions (*p *< .001) and for TB intentions (*p *= .003).

**Figure 2.**
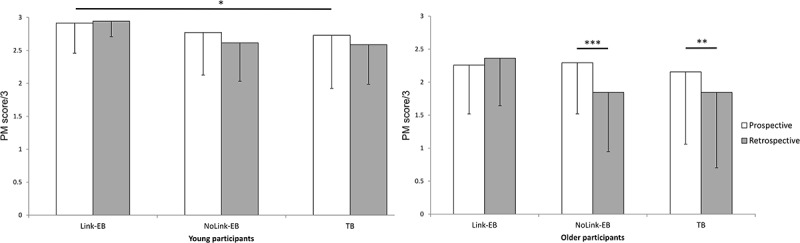
Recall of each component (prospective vs. retrospective) for each type of intentions (Link-EB vs. NoLink-EB vs. TB) among the young (left) and older (right) groups. * *p* < .05. ** *p* < .01. *** *p* < .001. Age effects highlighted on all PM measures are not depicted.

The main results are depicted in Figure 2.

### Cognitive functions subtending the age-related decline in PM

#### Effect of ageing on complementary measures

Results for the complementary cognitive tasks are reported in Table 2. Two-sample *t*-tests showed that young individuals performed better than their older counterparts on retrospective episodic memory, processing speed, inhibition, planning, and binding. No significant difference was found for shifting or time estimation.

**Table 2.** Young and older groups’ performances (mean ± standard deviation) on the complementary cognitive assessment.

Cognitive function	Test	Young	Older	*p*
Retrospective episodic memory				
Free recall	RL/RI-16	53.64 ± 5.07	45.00 ± 6.88	<.001
Processing speed	Stroop color	53.68 ± 6.25	58.09 ± 7.27	.003
Executive functions				
Shifting	TMT B-A	123.83 ± 67.51	120.51 ± 81.10	.91
Inhibition	Stroop interference	.61 ± .19	1.12 ± .54	<.001
Updating	Running span	34.80 ± 8.34	28.34 ± 7.77	.002
Planning	Zoo	6.84 ± 2.56	4.68 ± 3.50	.003
Binding	Original task	18.96 ± 0.98	15.09 ± 2.96	<.001
Time estimation	Original task	38.30 ± 46.14	37.51 ± 15	.94

#### Forward stepwise regressions

Forward stepwise regression analyses were performed for the total scores of Link-EB, NoLink-EB, and TB intentions, as well as for the total scores of the prospective and retrospective components of PM (Table 3). These analyses showed that the best predictors of PM recall were (1) binding for Link-EB intentions, (2) planning and retrospective memory for NoLink-EB intentions, (3) binding for TB intentions, as well as (4) binding and processing speed for the prospective component and (5) binding and retrospective memory for the retrospective component.

**Table 3.** Forward stepwise regressions on the different PM scores with complementary neuropsychological scores as predictors for the whole sample.

	Step	Predictors	*R*^2^	*F*	β	*p*
Link-EB	1	Binding	*0.32*	*28.70*	*0.56*	*<.001*
NoLink-EB	1	Planning	*0.20*	*16.68*	*0.46*	*<.001*
	2	Planning	*0.29*	*9.55*	*0.36*	*0.003*
		Retros. memory		*6.43*	*0.29*	*0.014*
TB	1	Binding	0.30	26.50	0.55	*<.001*
Prospective	1	Binding	*0.44*	*48.62*	*0.66*	*<.001*
component	2	Binding	*0.48*	*33.82*	*0.57*	*<.001*
		Processing speed		*6.26*	*−0.25*	*0.015*
Retrospective	1	Binding	*0.39*	*39.00*	*0.62*	*<.001*
component	2	Binding	*0.48*	*23.52*	*0.49*	*<.001*
		Retros. memory		*10.27*	*0.32*	*0.009*

#### Mediation analyses

**Figure 3.**
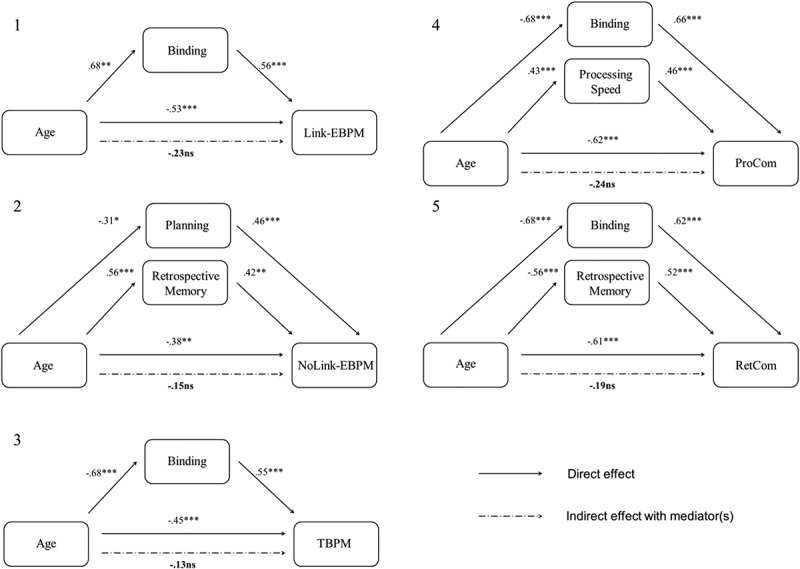
Mediation of the effect of ageing on prospective memory for (1) Link-EBPM intentions, (2) NoLink-EBPM intentions, (3) TBPM intentions, (4) the prospective component (ProCom), and (5) the retrospective component (RetCom). Coefficients correspond to the beta weights. *ns* = nonsignificant. * *p* < .05. ** *p* < .01. *** *p* < .001.

Mediation analyses were then performed for the same five total PM scores. For each PM measure, we simultaneously entered the best predictors as revealed by the forward stepwise regression analyses. Results are reported in Figure 3. The effect of ageing on PM was mediated by (1) binding for the recall of Link-EB intentions (CI = [−.0281, −.0026]), (2) planning plus retrospective memory for the recall of NoLink-EB intentions (CI = [−.0306, −.0046]), (3) binding for the recall of TB intentions (CI = [−.0235, −.0066]), (4) binding plus processing speed for the recall of the prospective component (CI = [−.0486, −.0086]), and (5) binding plus retrospective memory for the recall of the retrospective component (CI = [−.0601, −.0149]).

## Discussion

The present study is the first to have investigated the effect of ageing on PM, using a VR task to circumvent the methodological limitations of naturalistic and laboratory settings [28,31,33,34,85,86]. We found an age-related decline in PM performance for the recall of each component whatever the type of intention. However, when there was a weak link between the prospective cue and the retrospective component of intentions, the recall of the retrospective component was more challenging for older individuals. Finally, we showed that binding, processing speed, retrospective episodic memory, and, to a lesser extent, executive functions mediate the age-related decline of PM.

### A PM decline in ageing: using VR to circumvent the limitations of laboratory and natural settings

The first major finding of this study is that the recall of intentions suffers from an age-related decline in a VR task, which is thought to be more ecological than classic laboratory paradigms. This result extends those of previous studies conducted using more conventional tasks (see [27,28] for meta-analyses) or an ecological task in the laboratory [36]. Indeed, retrieval of both components of EB and TB intentions was less efficient in older participants, such that they were less accurate in carrying out the intention at the appropriate place (EB) or time (TB), and recalled fewer intentions than the young adults.

According to Philips and colleagues [33], several factors may influence the direction of age effects when investigating PM. First, the high level of control participants have over the task, insofar as they are free to plan the recall of intentions (e.g. by choosing specific cues to execute the task), may favour older individuals in naturalistic settings. In our VR task, the experimenter exerted full control over the target cues and times, which may also have decreased older adults’ performance [1,86]. Moreover, according to multiprocess theory [14,15], the level of attentional processes required to complete the ongoing task is one of the key points that determines the difficulty of the PM task, and affects older participants’ performance. For our VR task, we imposed an ongoing activity consisting in driving safely in the virtual city and exploring the environment. Even though driving may be regarded as quite an automatic activity, urban driving may require a high level of attentional resources, in order to drive, monitor the environment and distinguish relevant from irrelevant stimuli in the cluttered visual array to complete the PM task [87]. Thus, the decline in attentional resources in ageing [88,89] may have affected older adults’ PM performance, especially as they were less accustomed to this kind of equipment. Obviously, these statements do not mean that the deleterious effect of ageing we found on PM retrieval was only owing to the nature of the ongoing task. They rather do underlie the ecological validity of our task which placed the participants in a situation which seem as complex as those of everyday life.

### Effect of ageing on the recall of both components of intentions

#### Effect of ageing on recall of the prospective component according to the nature of the target

Overall, our results showed a deleterious effect of age on the recall of the prospective component of intentions, with no difference according to type of intention. Interestingly, while most studies showed that the recall of TB intentions was more sensitive to ageing than that of EB ones, two previous studies found the opposite result [12,13]. It has therefore been suggested that the characteristics of the PM task modulate the sensitivity of the recall of EB and TB intentions to ageing. In the present experiment for which we found no difference for the recall of the prospective component of EB and TB intentions in older individuals, they had to retrieve these intentions in parallel, requiring them to monitor both the virtual environment and the clock while driving in the virtual town. In other words, they had to shift constantly from one level of stimulus analysis to another, a situation constantly prone to an age-related impairment [90]. Overall, our results are in line with PAM theory [53,91], according to which the effect of ageing on the recall of the prospective component can be explained by older adults’ difficulty shifting between processes related to the ongoing task and processes related to the evaluation of responses to the stimuli in the environment (i.e. appearance of target places and times in this experiment). These processes prepare individuals for the return of the intended action to the focus of attention when the targets are encountered, and require the involvement of executive processes. We therefore suggest that older adults were disadvantaged for the recall of the prospective component of all types of intentions because they had difficulty detecting the target places and times, and/or reallocating them their prospective status, in other words in recognizing prospective cues for what they were, which may rely on retrospective memory abilities. This last proposition fits with the finding that young adults outperformed older individuals in post-test free recalls of intentions (see Appendix) which suggests that older individuals’ lower performance was more likely to be mainly explained by forgetfulness of intentions than by difficulty in detecting prospective cues or using VR equipment.

#### Effect of ageing on recall of the retrospective component according to the strength of the link between the prospective cue and the retrospective component

We found that young individuals outperformed their older counterparts on the recall of the retrospective components of Link-EB, NoLink-EB, and TB intentions. However, the recall of the retrospective component was more challenging for older individuals when there was a weak link between the prospective cue and retrospective component (i.e. for NoLink-EB and TB intentions). For these types of intentions, retrieval cannot rely on reflexive-associative processes and a strategic search in memory is required. Our results suggest that when young individuals correctly detected that something had to be done, they efficiently conducted the strategic search in memory for the retrospective component, and were able to perform the correct action. By contrast, in older adults, when the association was weak, the recall of the prospective component was not always followed by successful retrieval of the associated retrospective component. This result suggests an age-related decline in the controlled processes implemented to strategically search for the retrospective component in memory. This is in line with previous naturalistic studies of PM showing that even if ageing affects the recall of both components, the recall of the retrospective one is more sensitive to ageing [49,51]). Interestingly, the increase in the effect of ageing on the recall of unrelated intentions fits in with the effect of an age-related binding decline in older individuals on PM functioning, as it may reduce older individuals’ ability to retrieve the associated retrospective component.

### Cognitive functions mediating the effect of ageing on recall of the prospective and retrospective components

Overall, our results showed that PM performance is mainly predicted by binding, processing speed, retrospective memory, and planning scores. The decline in these cognitive functions may contribute to the age-related decline in PM. These results are consistent with previous studies underlying the involvement of these cognitive functions in PM functioning [13].

Binding was found to be the best predictor of the recall of both components, and one of the mediators of the effect of ageing on the recall of these components. This result extends the findings of a previous study in which an age-related binding decline accounted for a large proportion of the age-related decline in the recall of EB intentions [13]. In the field of retrospective episodic memory, it has been suggested that binding allows perceptual features to be integrated into single memory units [92,93]. Similarly, we hypothesized that binding provides the glue that holds the prospective component-retrospective component dyad together. The more efficient the glue at encoding, the fewer the controlled processes required for the retrieval of intentions. In the present experiment, the number of associations between cues/times and actions to perform was very high, compared with classic laboratory paradigms (i.e. one specific cue associated with one answer, repeated over time). The older individuals’ PM decline in our study may have stemmed from difficulty creating, maintaining, and retrieving such a large number of associations in memory. Interestingly, memorizing and executing such a list of intentions is closer than classic laboratory paradigms to real-life situations, where several EB and TB intentions must be simultaneously held in memory, and may interfere with each other. This interference may increase the complexity of binding the correct retrospective component to a given cue/time. Along the same lines, the increased number of cues/times may have made them less distinctive, making it harder to detect them quickly.

Processing speed was found to be the second best predictor of the effect of ageing on the recall of the prospective component. This result is in line with the general slowdown in ageing that has an impact on cognitive functions in general. In addition, it can be related to the high number of intentions participants had to retrieve and, by extension, to the high number of cues they had to detect. Indeed, the cognitive slowdown characterizing ageing may have reduced older individuals’ ability to fully explore the environment and reduced their ability to detect the targets at the correct moment. This is consistent with the contribution of the age-related processing slowdown to the age-related decline in memory [94,95], and with the importance of processing speed in PM functioning [96].

Retrospective episodic memory was found to be the second best predictor when modelling the effect of ageing on the recall of the retrospective component. In our study, older individuals were particularly disadvantaged for the recall of intentions with a weak link between the prospective cue and retrospective component. For these intentions, the difficulty of creating a sufficient strength of association to allow for reflexive-associative retrieval may have led to a need for controlled processes to actively search for the content of the intention in memory [14,15]. The age-related loss of efficiency of these controlled processes probably accounted for the poorer performance of the older individuals on the recall of the retrospective component for these intentions. This result is also consistent with studies showing that impaired retrospective episodic memory has a deleterious effect on the recall of the retrospective component [58,97].

### Cognitive functions mediating the effect of ageing on recall of different types of intentions: the specific case of nolink-eb intentions

While the deleterious effect of ageing on the recall of Link-EB and TB intentions was only mediated by binding decline, emphasizing the major role of binding in PM functioning, two other cognitive functions were found to mediate the recall of NoLink-EB intentions. Planning appeared to be the first predictor of the effect of ageing in this case, which is in line with studies demonstrating the involvement of executive functions in PM [56,57,98,99] and an age-related decline in these functions [100–102]. This is also consistent with a previous VR study in young individuals suggesting that following a predetermined plan is critical for the successful fulfilment of delayed intentions [47]. This finding might suggest that young adults were more able to plan the task fulfilment during encoding. Moreover, even though no explicit planning instructions were given to participants before retrieval, we can assume that they relied on this process to prepare for the detection of the prospective cues of NoLink-EB intentions [47,103]. Participants may have adopted such a strategy to overcome the apparent difficulty of retaining these intentions. Planning intentions was possible because they had seen the sequence in which the cues would appear during the familiarization phase. As planning for specific target events is supposed to obviate the need for strategic monitoring and improve PM under experimental conditions that require the involvement of controlled processes, its decline in ageing may have contributed to the older individuals’ lower performance [14,104]. By contrast, our results show that planning was not a major determinant of performance when participants could rely on automatic processes to recall intentions (i.e. for Link-EB intentions), or when the sequence of appearance of the targets within the environment could not be planified (i.e. for TB intentions).

Retrospective episodic memory was found to be the second predictor of the effect of ageing on the recall of NoLink-EB intentions, in line with a study conducted by Gonneaud et al. [13]. For these intentions, the weakness of the link between the prospective cue and the retrospective component led to an effortful search for the retrospective component in memory. This was not the case for the recall of Link-EB intentions, for which the reflexive-associative process was sufficient to automatically trigger the recall of the retrospective component, obviating the need for retrospective episodic memory processes. As we have already mentioned, this particularly effortful process reduced older adults’ ability to recall the intention [49,51]. For the recall of TB intentions, retrospective memory was not found to be a predictor of participants’ performance. This result does not mean that retrospective memory is useless when remembering these intentions, but, at least in our study, other functions were more able to account for the differences between young and older adults’ performances.

More generally, these results highlight the involvement of common (i.e. binding) and specific (i.e. executive functions, retrospective memory, and processing speed) cognitive functions when recalling different types of intentions. Implementing these various processes in a single task was certainly conducive to the increase in the deleterious effect of ageing on the recall of intentions.

## Limitations

VR provided a higher level of ecological validity than classic laboratory tasks that allowed us to mimic a real situation all the while blocking the compensation strategies implemented by individuals in daily life. However, our VR task gave rise to several limitations. Although the older participants mostly reported that the device was very pleasant to use, some of them felt uncomfortable during the experiment. More precisely, it was impossible for five older adults to complete the task because of motion sickness. We are hopeful that over the next few years, technological advances will result in more immersive material and environments to avoid these inconveniences. It would also be interesting to create tasks closer to daily life events, which means for example inserting various activities in a larger timescale.

Regarding the PM task, intentions to perform were exclusively provided by the experimenter, which is only part of what happens in real-life situations. Forthcoming studies could compare the effects of ageing on this type of intention and on intentions formed by the participants. Some intentions may also appear unnatural and future studies should use unrelated but more natural ones. Moreover, the ongoing task consisted in driving in the environment making it impossible to obtain a specific measure of participants’ performance in this task Thus, it was not possible to quantify whether the task was more demanding in terms of cognitive resources for older individuals than for young ones. Finally, when investigating cognitive substrates of PM, each cognitive construct was only measured with one single task which may have reduced the scope of our investigations. The use of more sensitive shifting and time-estimation abilities tasks might for example have shown the expected relation between these cognitive functions and PM that we surprisingly did not find.

## Conclusion

This study highlighted a deleterious effect of ageing on the recall of intentions, in line with previous evidence of PM decline in ageing. More specifically, using VR to improve ecological validity and experimental control, we highlighted a deleterious effect of ageing on the recall of both components of intentions, regardless of the nature of the intention and whatever the strength of the link between the prospective cue and the retrospective component. However, the difference between young and older individuals was more pronounced for the recall of the retrospective component when the relationship between the prospective cue and retrospective component was weak. Thus, the weakness of that link could be one of the reasons for the greater age-related decline when recalling TB intentions. Above and beyond the major role that the decline in binding seems to play in the age-related decline of PM under all conditions, the involvement of different cognitive functions according to the nature of the intention shows that the mechanisms subtending PM recall vary according to the characteristics of the intentions. Finally, the considerable flexibility of such environments should make it possible to explore a wide range of theoretical and clinical questions by manipulating large numbers of factors, thereby helping us to achieve a better understanding of complex cognitive functions such as PM. For example, as VR was found to be suitable for assessing PM in healthy ageing, it may also be relevant for this kind of assessment in other populations, notably patients with mild cognitive impairment or Alzheimer’s disease [72] (see also [105] for review).

## Appendix

**Table A1.** List of intentions.

Link-EB	NoLink-EB	TB
Buy a TV magazine at the newsstand	Buy glasses at the fountain	Call the restaurant to book a table after 2 minutes
Check the bus timetables at the bus shelter	Make an appointment with the dentist at the car park	Fill up with gas after 3 minutes
Buy a book of stamps at the post office	Buy a calendar at the city hall	Take a pill after 4 minutes

**Table A2.** Two-sample t-tests for participants’ performance in post-test free and cued recalls of intentions.

Post-test recall /3	Young	Older	*p*
**Free recall**			
Link-EB	2.66 ± 0.54	2.00 ± 0.76	<.001
NoLink-EB	2.56 ± 0.62	2.00 ± 0.80	.002
TB	2.63 ± 0.59	1.90 ± 1.05	<.001
**Cued recall**			
Link-EB	3.00 ± 0.00	2.83 ± 0.38	.010
NoLink-EB	2.76 ± 0.49	2.38 ± 0.78	.021
TB	2.71 ± 0.57	2.17 ± 0.85	.003
